# Are anaplastic lymphoma kinase (ALK) and O6-methylguanine-DNA methyltransferase (MGMT) promoter methylation driver biomarkers of pulmonary neuroendocrine tumors (NETs) and carcinomas (NECs)?

**DOI:** 10.18632/oncotarget.28240

**Published:** 2022-06-01

**Authors:** Birgitta Hiddinga, Karen Zwaenepoel, Annelies Janssens, Jan Van Meerbeeck, Patrick Pauwels

**Affiliations:** ^1^Department of Pulmonary Diseases, University Medical Center Groningen, Groningen, The Netherlands; ^2^Department of Pathology, Antwerp University Hospital, Edegem, Belgium; ^3^Department of Pulmonology and Thoracic Oncology, European Reference Network for Rare or Low Prevalence Lung Diseases: ERN-LUNG, Antwerp University Hospital, University of Antwerp, Edegem, Belgium

**Keywords:** MGMT promoter methylation, anaplastic lymphoma kinase, neuroendocrine tumor, neuroendocrine carcinoma, small cell lung cancer

## Abstract

Background: Novel targets in neuroendocrine tumors (NETs) and neuroendocrine carcinomas (NECs) are needed to improve outcome. The presence of O6-Methylguanine-DNA methyltransferase (MGMT) promoter methylation in NETs and NECs may act as a predictive marker for response on treatment with temozolomide. As anaplastic lymphoma kinase (ALK) plays an important role in the nervous system we hypothesized that ALK rearrangement can act as a biomarker in patients with NETs and NECs.

Materials and Methods: We performed a retrospective analysis to establish the frequency of MGMT promoter methylation and ALK expression in tissue samples of patients with NETs and NECs.

Results: 21% (14/67) of patients tested positive for MGMT promoter methylation. MGMT promoter methylation was present in 33% (3/9) patients with typical carcinoid, in 22% (2/9) patients with atypical carcinoid, in 22% (8/37) patients with small cell lung cancer and in 8% (1/12) patient with large cell neuroendocrine carcinoma. ALK- expression was present in 14% (10 of 70 patients). In all of these patients, no ALK-rearrangement nor ALK-mutation was revealed.

Conclusions: Routine testing of NET and NEC samples for an ALK rearrangement is not recommended as ALK-expression is not associated with an ALK-rearrangement. Routine testing of NET and NEC samples for MGMT will detect a promoter hypermethylation in a sizable minority of patients who are eligible for a targeted treatment with temozolomide.

## INTRODUCTION

Neuroendocrine tumors (NETs) and neuroendocrine carcinomas (NECs) are a subgroup representing less than 20% of lung tumors, with pulmonary NETs considered an orphan disease with an incidence of about 2% of all lung tumors [1–3]. NETs and NECs encompassing a morphologically and clinically distinct spectrum from typical carcinoid (TC) (grade 1) and atypical carcinoid (AC) (grade 2) tumors, to the highly aggressive neuroendocrine carcinomas (NECs) grade 3 and 4, small cell lung cancer (SCLC) and large cell neuroendocrine (LCNEC) variants with a high metastatic potential and a poor prognosis [4]. Their common phenotypic characteristic is the expression of features as neuroendocrine granules and the secretion of paraneoplastic cytokines and hormones, which reflect a common origin from the embryonal neuroendocrine crest. NETs arise from cells throughout the endocrine system. Although the different types of pulmonary NETs originate from the Kulchitsky cells of the bronchial mucosa, different mutations cause different biology and they are therefore considered separate clinical entities [5].

In the treatment of patients with advanced non-small cell lung cancer (NSCLC), a paradigm shift occurred over the last years by the discovery of actionable driver mutations and translocations susceptible for targeted treatment. Despite extensive research, few innovations in the treatment of NETs and NECs have been proposed. New potential targets in NETs and NECs are needed to improve outcome.

In NETs, DNA-promoter methylation might be a mechanism that maintains the neuroendocrine biology [6]. DNA-promoter methylation is a well-known epigenetic process and refers to one of the major mechanisms for silencing tumor suppressor genes. The DNA repair protein encoded by the O^6^-Methylguanine-DNA methyltransferase (MGMT) suppressor gene removes alkyl groups from the O^6^ position of guanine [7]. The epigenetic silencing of the MGMT gene via promoter methylation of specific CpG islands of its promoter leads to loss of expression of MGMT enzyme [8]. MGMT promoter methylation status can be assessed by polymerase chain reaction (PCR) on either a cytology specimen from (needle) aspirations or a tissue specimen from biopsies [9]. Temozolomide has shown beneficial effects in patients with relapsed SCLC, especially in a subgroup associated with the presence of the MGMT promoter methylation [10]. The drug has shown promising activity in patients with glio(-blast-)oma and relapsed SCLC, with a response rate of 22% in all comers, of 19% in third line and of 38% in patients with brain metastases [10]. It is hypothesized that the presence of MGMT promoter methylation in NETs and NECs may act as a predictive marker for response to treatment with temozolomide [11].

The ALK fusion gene is mostly formed by a rearrangement occurring on the short arm of chromosome 2 and involves the genes encoding for ALK (2p23.2) and echinoderm microtubule-associated protein-like 4 (EML4) (2p21) [12]. Several other translocation partners have been described. Rearrangement of ALK occur in a variety of tumors, including NSCLC, anaplastic large cell lymphomas, inflammatory myofibroblastic tumors and neuroblastomas [13–15]. Little is known about ALK rearrangement in NETs and NECs [16]. As ALK plays an important role in the development of the brain and in specific neurons in the nervous system, we hypothesize that ALK expression or translocation is present in tumors of the neuroendocrine crest [17]. ALK rearrangement can act as biomarker for the treatment with ALK-inhibitors in this selected patient group. Repurposing of drugs used for other indications and/or tumor types is an acceptable and innovative strategy in the advancement of treatment of these devastating carcinomas. We performed a retrospective analysis on tissue samples of patients with NETs and NECs to establish the frequency of MGMT promoter methylation and the frequency of ALK expression and rearrangement.

## RESULTS

### Patients and tumor classification

After approval by the local Scientific board of the local Biobank and of the Ethical Committee and having obtained the consent of the patients, we collected from the local biobank the archival samples of 74 treatment-naïve patients who were diagnosed as NETs and NECs between January 2014 and December 2016, data of the hospital electronic system were retrospectively collected. Their characteristics such as age stage, diagnosis, performance score and treatment were extracted from their medical records. In case of surgically removed NETs, the primary tumor was tested for ALK and MGMT promoter methylation. In case of metastasized SCLC or LCNEC most samples were from metastases, either lymph node samples or metastases in other organs (except brain and bone metastases). Pathological diagnoses of these 74 patients were verified from archival tissue and made according to the World Health Organization classification based on morphology [4]. Confirmation of the pathologic diagnosis was made by a dedicated pathologist (PP) and was performed on IHC with synaptophysin, chromogranin A, and Ki-67. Tumors were classified as NETs, typical carcinoid (grade 1) and atypical carcinoid (grade 2) tumors, to the NECs grade 3 and 4, SCLC and LCNEC variants with a high metastatic potential and a poor prognosis.

Most patients had metastatic SCLC. Patients characteristics and test results are described in Table 1. There was adequate tissue available for ALK testing in 70 patients and in 67 patients for MGMT promoter methylation testing. Patients were treated according to the guideline. Patients were not treated with an ALK-inhibitor in case of positive ALK-IHC, nor with temozolomide if presence of MGMT promoter methylation as both treatments were not available as reimbursed medication.

**Table 1:** Patient characteristics and results of ALK and MGMT testing

	Total: *N* = 74	ALK IHC positive	ALK IHC negative	MGMT positive	MGMT negative
Evaluable cases		10	60	14	53
Age range (years)	39–88	46–88	39–88	45–88	39–88
Sex					
Male	40 (54%)	4	36	7	26
Female	34 (46%)	6	28	7	27
Disease stage					
I	17	3	13	4	11
II	7	0	6	2	4
III	20	3	16	4	15
IV	30	4	25	4	23
Tumor histology					
Typical carcinoid	10	2 (22%)	7 (78%)	3 (33%)	6 (67%)
Atypical carcinoid	9	2 (25%)	6 (75%)	2 (22%)	7 (78%)
SCLC	41	6 (15%)	33 (85%)	8 (22%)	29 (78%)
LCNEC	14	0 (0%)	13 (100%)	1 (8%)	11 (92%)

Abbreviations: ALK: anaplastic lymphoma kinase; IHC: immunohistochemistry; MGMT: O^6^-Methylguanine-DNA methyltransferase; NET: neuroendocrine tumor; NEC: neuroendocrine carcinoma; SCLC: small cell lung carcinoma; LCNEC: large cell neuroendocrine carcinoma.

### Analysis of ALK IHC, FISH and ALK mutations

**Figure 1:**
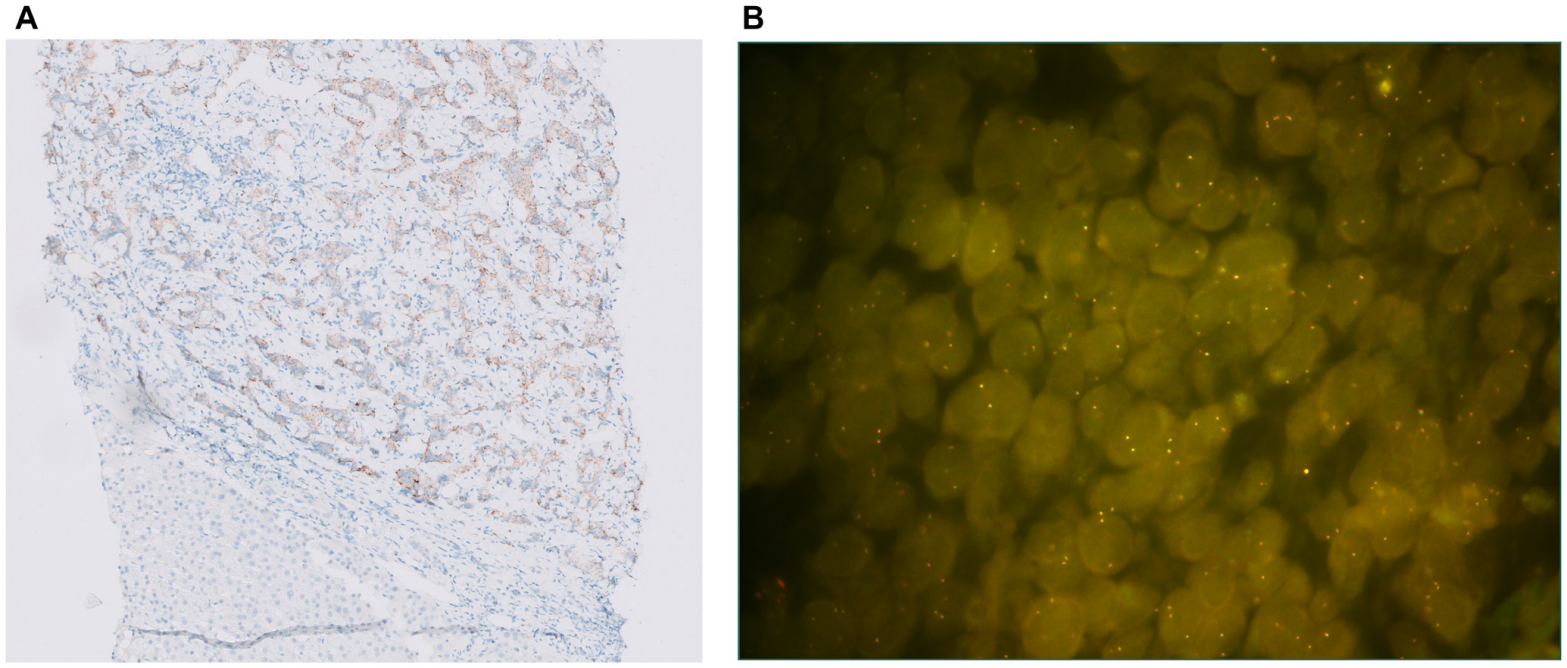
(**A**) ALK IHC staining (10X): moderate to strong staining in 70% of the tumor cells. (**B**) ALK FISH: ALK IHC positive SCLC sample without ALK rearrangement (only fused signals present).

Ten of 70 (14%) specimens were ALK IHC positive (Table 1). The ten ALK IHC positive specimens consisted of two typical carcinoids, two atypical carcinoids, and six SCLC. None of the 13 LCNECs were ALK IHC positive. ALK IHC positive specimen were tested for ALK FISH (Figure 1). None of them showed rearrangements. In 5 tissues of high ALK expression the presence of ALK mutations was tested, but no ALK mutations were present.

### Analysis of the MGMT promoter methylation testing

In 67 of 74 patients, tissue was sufficient for evaluation. In 21% (14/67) of patients tested positive for MGMT promoter methylation (Table 1). MGMT promoter methylation was present in 33% (3/9) patients with typical carcinoid, in 22% (2/9) patients with atypical carcinoid, in 22% (8/37) patients with SCLC and in 8% (1/12) patient with LCNEC.

## DISCUSSION

This is one of the largest series with NET and NEC where the role of MGMT promoter methylation and ALK is studied.

In our series we found ALK expression in 14% of a cohort of 70 patients with NETs and NECs. No ALK- rearrangement was present. These findings are in accordance with the largest series of patients with SCLC published [18–20] (Table 2). To evaluate whether ALK expression in NET/NEC is generally associated with ALK mutations we selected five patient samples with a high ALK expression for ALK mutation analysis. We made the assumption that if ALK-mutations were absent in the high-expressors, patients with a low or negative ALK-expression harbored no ALK-mutations. None of the specimens with a high ALK expression ALK mutations were detected. Kondoh et al., examined specimens of 142 patients with SCLC, 41 patients with LCNEC and 11 patients with carcinoids [18]. In the SCLC cohort, ALK expression was detected in 16 of 142 (11.3%) and 4 of 12 specimens were found to carry copy gain numbers. In the LCNEC and carcinoid cohort no rearrangements, no amplifications, no point mutations and no ALK expression was found. No significant association was found between ALK-expression and overall survival. The authors conclude that ALK-expression in SCLC was due to intrinsic expression of a normal ALK-transcript.

**Table 2:** Studies on ALK in NETs and NECs

		Samples (*n*)	ALK expression	ALK rearrangement	ALK mutation, amplification, copy gain number (CGN)	Response on ALK inhibitor
Kondoh [18]	SCLC	142	16 (11.3%)	Not present	CGN: 4/12 (33, 3%)	No response of crizotinib in *in-vitro* cell lines
	LCNEC	41	0	Not present	Not present	
	Typical carcinoid	11	0	Not present	Not present	
Nakamura [19]	SCLC	69	2 (2.9%)	Not present	NA	NA
	LCNEC	106	1 (0.9%)	Not present		
	Carcinoid	52	0	Not present		
Karlsson [20]	LCNEC	32	NA	Not present	NA	NA
Toyokawa [16]	SCLC and adenocarcinoma	1	Present	Variant 1 EML4 – ALK fusion	NA	NA
Toyokawa [21]	SCLC and adenocarcinoma	1	Present	Variant 2 EML4 – ALK fusion	NA	NA
Bai [22]	SCLC and adenocarcinoma	1	Present	KLC1 – ALK fusion	NA	NA
Pronk [23]	SCLC and atypical carcinoid	1	NA	NA	NA	NA
Wang [24]	Atypical carcinoid	1 (liquid biopsy)	NA	SMC5 – ALK fusion	Not present	Alectinib, partial response
Nakajima [25]	Atypical carcinoid	1	Present	Present	NA	Crizotinib, partial response
Fukuizumi [26]	Atypical carcinoid	1	Present	Variant 3a/b EML4 – ALK fusion	Not present	Crizotinib, no response
Omachi [27]	LCNEC	1	Present	Variant 2 EML4 – ALK fusion	NA	Crizotinib, progressive disease
Miyoshi [28]	SCLC	1	Present	Not present	NA	NA
Hayashi [29]	LCNEC	1	NA	Present	NA	Alectinib, response

Abbreviations: ALK: anaplastic lymphoma kinase; NET: neuroendocrine tumor; NEC: neuroendocrine carcinoma; SCLC: small cell lung carcinoma; LCNEC: large cell neuroendocrine carcinoma; NA: not analyzed.

In another series aberrant ALK-expression in 227 pulmonary NECs was observed in 2 (2.9%) of 69 SCLC and 1 (0.9%) of 106 LCNEC [19]. In 52 carcinoid tumors no ALK-expression was observed. In three ALK positive NECs no ALK rearrangement nor amplification was found, also no ALK-mutation was detected. In a smaller series of 32 LCNEC tumors, no ALK-expression was seen. Nor were ALK-fusions or ALK-mutations detected [20]. This data is in agreement with our results demonstrating that ALK expression is not associated with the presence of an ALK-rearrangement or ALK-mutation.

A number of case-reports on ALK-rearrangements in atypical carcinoid, SCLC and LCNEC have been reported (Table 2). Not all specimen were tested for ALK-expression, and no ALK-mutations were revealed.

Two cases of SCLC were reported containing an ALK-rearrangement in a series of 30 patients with SCLC [16, 21]. Both cases had a combined SCLC with an ALK-expression and an adenocarcinoma. In the first case presented the adenocarcinoma component harbored an EGFR-mutation, deletion in exon 19 [16]. It was stated by the authors that adenocarcinomas with an EGFR-mutation can transform into SCLC in the process of acquiring resistance to EGFR tyrosine kinase inhibitors. As this patient didn’t receive any medication before diagnosis, the mechanism rather reflects coincidence than transformation as acquired resistance. In the second case report a patient with SCLC harboring a variant 2 of the EML4-ALK fusion gene [21]. This SCLC was repeatedly confirmed by histological biopsy, however stained negative for TTF1 and positive for ALK. The patient showed a partial response on chemotherapy. After progression, a biopsy confirmed SCLC with ALK-expression. The patient didn’t receive treatment with an ALK-inhibitor. In another case of combined SCLC and adenocarcinoma, an ALK gene alteration was found in both components [22]. In a case report of combined SCLC and atypical carcinoid no testing for ALK was conducted [23]. These patients received standard of care, none of these patients were treated with ALK-inhibitors. We hypothesize that these findings in combined SCLC and adenocarcinoma or a combined SCLC and atypical carcinoid suggests that the origin of the lung tumor may be monoclonal. We did not include combined tumors in our series.

Case reports of NETs or NECs that were treated with ALK-inhibitors showed different responses. One case of atypical carcinoid with an ALK-rearrangement showed partial response on alectinib after progression on temozolomide and capecitabine [24]. Another patient with an atypical carcinoid with ALK-expression and ALK-rearrangement progressed after chemotherapy and was successfully treated with crizotinib [25]. An atypical carcinoid with variant 3a/b ALK-rearrangement did not respond to crizotinib [26]. Crizotinib as first generation ALK-inhibitor maybe less powerful. A case report of a patient with LCNEC with ALK rearrangement responding to alectinib after progression on chemotherapy [27]. In a case of advanced LCNEC expressing ALK on IHC and an ALK-rearrangement with FISH, the patient treated with crizotinib in first line [28]. The first evaluation six weeks later showed progressive disease. The conclusion is that ALK-rearrangement may not be of practical importance in LCNEC and that neuroendocrine tumors with ALK-rearrangement may be less responsive to ALK-inhibitors. This stresses the importance to assess ALK fusion genes with FISH or NGS (RNA) in case of ALK-expression [29].

In our series standard testing for ALK was done by FISH testing, as it was – at that time – the standard test considered ‘gold standard’. In later times we tested ALK IHC as the abnormal ALK protein product of fusion genes may be associated with elevations in ALK protein, detectable by IHC. A positive ALK-expression is considered sufficient indication for treatment with an ALK-inhibitor in NSCLC. Currently, superior second and third generation ALK-inhibitors are available with a better systemic and intracranial efficacy than crizotinib, which was used in some of the patients in the case reports. As sporadic cases of TKI-addicted ALK-altered lung cancers are in the NET/NEC population, selected patients fit enough for advanced line therapy, should be tested for the presence of ALK protein.

### MGMT promoter methylation in NETs and NECs

Epigenetic alterations in cancer are a potential source of predictive therapeutic biomarkers for personalized cancer treatment. Whereas MGMT promoter methylation may have predictive value, MGMT expression by IHC does not [30].

A feasibility study was conducted in relapsed SCLC to evaluate the MGMT promoter methylation in tissue, cytology and sputum [9]. Of 56 patients with SCLC, 30 tissue biopsies, 17 fine-needle aspirates, 8 bronchial washings and 1 sputum were available. Methylation analysis was obtained in 54 samples (and failed in two bronchial washings). MGMT promoter methylation was detected in 35.2% without any significant difference between histological and cytological samples (37.9% vs. 32%) (Table 3). The assay used for MGMT analysis is an in house developed validated assay for glioblastoma samples. The assay is highly suitable for glioblastoma samples as annual EQA schemes for central nervous system tumors demonstrate good results. Although the assay works well for small tissue fragments and cytology, the assay has not been validated on SCLC/NET/NEC samples. The degree of MGMT methylation is a continuous value and the ideal cut-off value for hypermethylation of SCLC/NET/NEC might be different than in central nervous system tumor. Another limitation is that when a partial loss of both chromosomes 10 occurs, the MGMT assay can produce a false negative result because this loss is not taken in account.

**Table 3:** Studies on MGMT promoter methylation in NETs and NECs

		Samples histology (*n*)	Samples cytology (*n*)	MGMT promoter methylation (%)	Response on temozolomide
Miglio [9]	SCLC	30	24	35.2	NA
Pietanza [10]	SCLC	27		48	RR 38%
Zauderer [37]	SCLC	8		87.5	1 PR (14%)
Walter [11]	Carcinoid	5		80	NA
Pietanza [39]	SCLC	32		31	Not significantly
Lei [31]	Carcinoid and LCNEC	12		16.6	NA
Lu [38]	SCLC	33		51.5	NA

Abbreviations: MGMT: O^6^-Methylguanine-DNA methyltransferase; NET: neuroendocrine tumor; NEC: neuroendocrine carcinoma; SCLC: small cell lung carcinoma; LCNEC: large cell neuroendocrine carcinoma; NA: not analyzed; RR: response rate; PR: partial response.

No prospective data about the incidence of MGMT promoter methylation is available in lung NETs. In other retrospective series, MGMT is methylated in 0–27% of lung NETs [31, 32]. This outcome is comparable to our series.

To our knowledge, one report is available describing MGMT promoter methylation in LCNEC samples. This study revealed the presence of MGMT promoter methylation in 2 of 6 patients [31]. Our retrospective series contains a larger patient group, but in only 1 patient out of 12 (9%) MGMT promoter methylation was detected.

MGMT promoter methylation is significantly associated with tumor response to temozolomide in glioblastoma multiforme and NETs [10]. Recent guidelines recommend temozolomide treatment in advanced unresectable progressive pulmonary atypical carcinoid tumors [33]. The optimal dosing regimen and schedule with temozolomide is still under debate [34]. Treatment with temozolomide is an option in relapsed advanced SCLC [35], however, the only approved second line treatment in relapsed SCLC is topotecan [36].

The efficacy of temozolomide was reported in several studies (Table 3). The sample size is too small to estimate a pooled response rate on temozolomide in the MGMT promoter methylation positive patients. Pietanza et al., studied 64 patients with progressive SCLC after one or two prior chemotherapy regimens who received temozolomide at 75 mg/m^2^ daily for 21 days of a 28-day cycle [10]. The primary endpoint was response rate. The tumor response of 22% was observed in an unselected group, in third line the tumor response was 19%. In those with brain metastases the tumor response was 38%. In 48% (*n* = 27) of patients, a MGMT promoter methylation was detected. The response rate to temozolomide was 38% in the MGMT promoter methylated group versus 7% in the group without MGMT promoter methylation, suggesting that a tumor response due to temozolomide may be associated with the presence of MGMT promoter methylation. Twenty-five patients were enrolled in a single center trial of a 5-day dosing regimen of temozolomide 200 mg/m2 in a 28-day cycle [37]. The rationale for this shortened dosing schedule was to avoid prolonged myelosuppression. The primary endpoint, tolerability, was met with common toxicity criteria grade 3 and 4 toxicity in 5 out of 25 patients. Temozolomide was well-tolerated. Responses were seen in 12 patients (48%, 95% CI: 3–31%). No responses in the brain were seen with this regimen. Eight tissues were tested for the MGMT promoter methylation and of these, 7 had evidence of promoter methylation of whom 1 had a partial response. The small sample size does not allow to draw solid conclusions about the predictive value of MGMT promoter methylation.

In another study, 17 out of 33 Chinese SCLC patients (51.5%) had MGMT promoter methylation [38]. A comparative study between temozolomide and veliparib versus temozolomide with placebo in patients with relapsed SCLC did not show improved progression free survival [39]. Analysis of MGMT promoter methylation as a biomarker was limited, as sufficient DNA was available in only 32 of 104 tumor samples. The MGMT promoter was methylated in 31% (7 of 32) of the samples tested and was not associated with tumor response or with improved progression free survival or overall survival.

Our series revealed MGMT promoter methylation in 22% of patients with SCLC. As SCLC is a recalcitrant illness, there is an unmet need for treatment options in relapsed or refractory disease. As guidelines recommend treatment with temozolomide, stratification by MGMT promoter methylation can select a patient group that benefits from temozolomide. We propose a prospective study in which a biomarker selected patient group with MGMT promoter methylation is treated with temozolomide.

## MATERIALS AND METHODS

### Neuroendocrine protein expression

Confirmation with immunohistochemistry (IHC) was performed with neuroendocrine markers such as synaptophysin, chromogranin A. Ki-67 expression was used as proliferation marker. IHC was performed with synaptophysin (clone DAK-SYNAP, RTU, Agilent), chromogranin A (Clone LK2H10, 1/500, Menarini), and Ki-67 (Clone MIB-1, RTU, Agilent) on an Autostainer Link 48 instrument (Agilent) using the Envision Flex detection kit (Agilent).

### ALK Immunohistochemistry

Subsequently, these samples were analyzed for ALK expression. FFPE sections (5-μm thickness) were stained using ALK 5A4 (Leica) with EnVison Flex+, mouse high pH detection reagents on an Autostainer Lin 48 instrument (Dako, Glostrup, Denmark). The sections were subsequently incubated in high pH buffer (20 min, 97°C; PT-Link, Dako), peroxidase blocking buffer (5min), primary antibody (1:50, 30 min), mouse-enhanced polymer-based linker (30 min), mouse secondary antibody (20 min), diaminobenzidene (5 min) and haematoxylin (5 min) as previously described [40]. ALK expression was assessed independently by one pathologist (PP) and one scientist (KZ). IHC ALK positive samples were evaluated with Fluorescence *in-situ* hybridization (FISH). High ALK- expressors were analyzed with next generation sequencing to detect ALK mutations.

### ALK Fluorescence *in-situ* hybridization

FISH was performed on 5-μm formalin-fixed, paraffin-embedded (FFPE) tissue sections using the Vysis ALK dual-color, break-apart rearrangement probe in combination with the Vysis pre-and post-treatment kit IV (Abbott Molecular, Des Plaines, IL, USA) according to the manufacturer’s instructions. Results were analyzed using a fluorescent BX41 microscope (Olympus, Center Valley, PA, USA) and evaluated according to the Vysis LSI ALK Probe manufacturer’s enumeration guidelines.

Fluorescence *in-situ* hybridization (FISH) with Vysis/Abbott LSI ALK probe was performed in IHC positive cases. ALK FISH was considered positive if at least 15% op tumor cells showed rearrangement (50 nuclei were evaluated).

### ALK mutation analysis

ALK mutation analysis was performed using an in house developed and validated Next Generation Sequencing panel detecting single nucleotide variations and small indels in genomic DNA of amongst other exon 22, 23 and 25 of the ALK gene with a sensitivity of 3%. From each sample 10 unstained slides of 5 μM thickness were prepared. Upon macro dissection of the tumor region gDNA extraction was performed using the QIAamp DNA Mini Kit (Qiagen) on a Qiacube instrument. Upon HaloPlex enrichment (Agilent) of the target DNA sequencing analysis was executed on a MiSeq platform using the MiSeq Reagent kit V2 (300 cycles) (Illumina). Analysis of the data was performed using the JSI SeqNExt v4.1.2 software.

### Methylation specific PCR of the promoter region of MGMT

MGMT promoter methylation was analyzed with PCR. Upon macro dissection, DNA isolation of FFPE section was performed using the QIAamp DNA blood mini kit (Qiagen). Bisulfite-mediated conversion of the extracted gDNA was performed using the EpiTect Bisulfite kit (Qiagen) according to the manufacturer’s instructions. TaqMan qPCR assay was performed on this converted gDNA to amplify MGMT and ACTB with the following primers and probes: MGMT-FWD 5′-GCGTTTCGACGTTCGTAGGT-3′, MGMT-REV 5′-GCACTCTTCCGAAAACGAAACG-3′, MGMT-PROBE 5′-FAM-CGCAAACGATACGCACCGCGA, ACTB-FWR 5′-TGGTGATGGAGGAGGTTTAGTAAGT, ACTB-REV 5′-AACCAATAAAACCTACTCCTCCCTTAA-3′, ACT-PROBE 5′-VIC-ACCACCACCCAACACACAATAACAAACACATA-3′. These primer and probe sequences were obtained from Parella et al., and Esteller et al., [41, 42]. Amplification was performed using the LightCycler 480 probes master mix (ROCHE) on a Cobas 4800 platform with an hybridization temperature of 60°C. In each run, a non-template control, a WT control and a positive control (2.5% U87D cell line in background of tonsil FFPE tissue) was included. The MGMT assay has been validated on glioblastoma samples with a detection limit of 1%. The assay is suited for both tissue and cytology samples and required a ratio of neoplastic cells of minimally 10%. The assay is ISO15189 accredited and annual participation to EQA scheme (GENQA CNS schemes) consistently demonstrated good results.

Samples were deemed informative if the Cp value of the ACTB gene was <31 and the samples were scored positive when for MGMT a Cp value of <36 was obtained. These Cp values were established and verified by respectively comparing the results with the assay described by Esteller et al., [42] and with other Belgian and Dutch diagnostic, accredited laboratories performing MGMT analysis in routine practice.

## CONCLUSIONS

A subset of NETs and NECs stains positive for ALK-IHC. As protein expression of ALK is especially found in neuronal tissue like thalamus, hypothalamus, midbrain and dorsal root ganglia, the question is whether altered ALK present in neuroendocrine tumors of the lung, can act as a target for treatment with ALK inhibitors. Although 14% of patients expressed ALK, rearrangement was absent. Since mutations in ALK tyrosine kinase domain have also be described to cause ALK expression, also ALK mutation analysis was performed. However, no ALK mutations were found. We suggest that ALK expression reflects the origin of the tumor, the neuroendocrine crest. In absence of an ALK-rearrangement there’s no indication for treatment with an ALK-inhibitor.

A sizable fraction of patients NEC and NET present with a MGMT promoter hypermethylation, which is considered a driver alteration for targeted treatment with temozolomide. Prospective data are needed, preferably in a randomized design. We hence recommend testing refractory or relapsing patients with NEC and NET for the presence of this alteration on archival tissue, in order to ascertain their eligibility for such a treatment.

## Abbreviations

AC: atypical carcinoid
ALK: anaplastic lymphoma kinase
IHC: immunohistochemistry
LCNEC: large cell neuroendocrine carcinoma
MGMT: O6-Methylguanine-DNA methyltransferase
NEC: neuroendocrine carcinoma
NET: neuroendocrine tumor
PCR: polymerase chain reaction
SCLC: small cell lung cancer
TC: typical carcinoid
